# Variations in Plankton Community Structure Between Freshwater and Saline–Alkaline Waters and Their Correlation with Nutrient Composition in *Macrobrachium nipponense*

**DOI:** 10.3390/ani16111591

**Published:** 2026-05-23

**Authors:** Shubo Jin, Zhenghao Ye, Hongtuo Fu, Yiwei Xiong, Hui Qiao, Wenyi Zhang, Sufei Jiang

**Affiliations:** 1 Wuxi Fisheries College, Nanjing Agricultural University, Wuxi 214081, China; 2 Key Laboratory of Freshwater Fisheries and Germplasm Resources Utilization, Ministry of Agriculture and Rural Affairs, Freshwater Fisheries Research Center, Chinese Academy of Fishery Sciences, Wuxi 214081, China

**Keywords:** *Macrobrachium nipponense*, saline–alkaline water resource, ionic compositions, plankton community, nutrient components

## Abstract

Essential amino acids and unsaturated fatty acids are key nutritional indicators. The human body preferentially absorbs these compounds, which have beneficial effects on health. In aquatic ecosystems, plankton communities serve as the primary food source for aquatic organisms, playing a crucial role in shaping their nutritional composition. In this study, we collected populations of *Macrobrachium nipponense* and corresponding water samples from ten distinct geographical locations across China. These sites included five freshwater resources and five saline–alkaline water resources. This study measured the ionic composition and plankton community structure of water samples, and analyzed the nutritional components of *M. nipponense*, aiming to identify indicator taxa linked to the nutritional value in this species. The results show significant differences in both nutritional components and plankton community structures between freshwater and saline–alkaline environments. This suggests a correlation between specific plankton taxa and the nutritional value of *M. nipponense*. Using relative sequence abundance data from metabarcoding, linear discriminant analysis effect size (LEfSe) analysis identified six plankton indicator taxa at the genus level. Their abundance differed significantly between the two habitat types. The saline–alkaline region had three associated taxa: *Cyclotella*, *Brachionus*, and *Chaetoceros*. In contrast, *Arctodiaptomus*, *Cryptomonas*, and *Limnoithona* were identified as indicator taxa for freshwater regions. Redundancy analysis (RDA) and Pearson correlation analysis revealed that, with the exception of the SY site, the abundance of *Chaetoceros* and *Brachionus* in saline–alkaline waters tracked with levels of K^+^, Ca^2+^, and HCO_3_^−^. Meanwhile, at the SZ site, plankton community richness rose with CO_3_^2−^. Furthermore, the potential correlations between plankton indicator taxa and the formation of specific nutritional components in *M. nipponense* were explored. These findings highlight the complex interactions among ionic composition, plankton indicator taxa, and nutritional value in *M. nipponense*. Ultimately, this study can support the development of artificial techniques to regulate the nutritional components of this commercially important species.

## Simple Summary

This study analyzed how plankton communities and water ions affect the nutritional value (essential amino acids and unsaturated fatty acids) of Macrobrachium nipponense. Ten sites across China were sampled—five freshwater and five saline–alkaline. Clear differences were found between the two habitat types, both in plankton communities and in prawn nutrition. Using metabarcoding and LEfSe analysis, six plankton indicator genera were identified at the genus level. In saline–alkaline waters, the indicators were *Cyclotella*, *Brachionus*, and *Chaetoceros*. In freshwater, they were *Arctodiaptomus*, *Cryptomonas*, and *Limnoithona*. RDA and Pearson correlation showed that, except for the SY site, the abundance of Chaetoceros and Brachionus in saline–alkaline waters tracked with K^+^, Ca^2+^, and HCO_3_^−^ levels. At the SZ site, plankton richness was found to increase with CO_3_^2−^. These findings point to possible links between plankton indicator taxa and the nutritional components of M. nipponense, highlighting complex interactions that could guide artificial regulation of nutritional quality for commercial use in this species.

## 1. Introduction

The oriental river prawn (*Macrobrachium nipponense*) is a commercially valuable freshwater species in China, known for its delicate texture, pleasant taste, and richness in micronutrients [1]. It has become a popular species in Chinese aquaculture, with annual production exceeding 223,000 metric tons in 2024. Major producing regions included Jiangsu, Anhui, Zhejiang, and Jiangxi. Each contributes over 20,000 metric tons per year, generating notable economic benefits [2].

Plankton communities are ubiquitous in aquatic ecosystems, where they serve as fundamental primary producers and contribute to the maintenance of ecosystem structure, functionality, and stability [3]. Elevated levels of salinity and alkalinity in aquatic environments impose considerable physiological stress on aquatic organisms, including both zooplankton and phytoplankton communities [4]. For example, a broad range of primary producers (such as cyanobacteria, dinoflagellates, and green algae) proliferate in low-salinity alkaline lakes, thereby supporting high primary productivity and the subsequent deposition of organic-rich mudstones. Hypersaline alkaline lakes, in contrast, are capable of sustaining only a specialized haloalkaliphilic green alga [5]. Under altered carbonate chemistry conditions in harbor water bodies, a moderate pH-equilibrated ocean alkalinization approach may enhance phytoplankton resilience and stimulate diatom activity [6]. Ocean alkalinity is also correlated with zooplankton standing stock and community composition in the Eastern Mediterranean Sea [7]. These chemical stressors induce osmotic and ionic imbalances due to high ion concentrations, potentially disrupting cellular homeostasis [8]. The progressive rise in salinity and alkalinity triggers adaptive osmoregulatory mechanisms in aquatic organisms and microorganisms, thereby giving rise to acute physiological challenges [9,10].

In natural water bodies, plankton communities serve as the primary food source for aquatic organisms, and their abundance and diversity strongly influence both the productivity and nutritional composition of higher trophic levels [11]. These microorganisms act as reservoirs of essential nutrients and play a key role in sustaining larval production in aquaculture systems [12]. The nutritional quality of aquatic organisms can be improved through the provision of live feed, given that the nutrient profiles of these plankton species are effectively transferred and accumulated in the consumers [13,14]. Comparative studies have shown that, for fish larvae and early post-larvae, using zooplankton as a dietary source led to better nutritional outcomes than using artificial feed formulations [15,16]. Supplement of specific plankton taxa (such as *Chaetoceros gracilis*, *Chaetoceros muelleri*, *Pavlova lutheri*, *Brachionus plicatilis*, and *Artemia franciscana*) in the diet can affect the nutritional quality of *Ucides occidentalis* larvae, mainly by promoting the accumulation of key long-chain polyunsaturated fatty acids in their tissues [15]. Likewise, supplementing the diet with copepods (*S. poplesia*) improves the survival, growth, and nutritional composition of *Paralichthys olivaceus* larvae and juveniles [16]. This phenomenon can be attributed to the central position of zooplankton in aquatic food webs, where they facilitate the transfer of chemical energy from autotrophic phytoplankton to higher trophic levels, including macroinvertebrates and fish [17,18,19]. As a major component of the nutritional intake of aquatic organisms, zooplankton communities also contribute significantly to nutrient cycling and energy flow within aquatic ecosystems [20,21]. Their distribution patterns and population dynamics, in turn, exert regulatory effects on overall ecosystem productivity.

Amino acids play essential roles in cell growth, tissue repair, and metabolic regulation [22]. They fall into two categories: essential amino acids (EAAs) and non-essential amino acids (NEAAs) [22]. Animals cannot de novo synthesize EAAs and therefore must obtain them from dietary protein. The composition and concentration of EAAs largely determine the nutritional value of food proteins [23,24,25]. Fatty acids carry out a range of biological functions, such as energy storage, serving as structural components of cell membranes, and regulating physiological processes [26]. They are divided into saturated fatty acids (SFAs) and unsaturated fatty acids (UFAs) [27]. UFAs are known to benefit human health. They have positive effects on the regulation of blood lipids and immune function [28,29,30]. With these nutritional goals, a long-term objective in *M. nipponense* aquaculture is to optimize its nutrient profile by raising the levels of EAAs and UFAs, thereby improving the nutritional value of this commercially important species.

DNA metabarcoding has become a standard tool for identifying plankton in natural aquatic environments. The method sequences a short region of the mitochondrial genome, chosen for its high variability across species and the existence of extensive reference databases [31]. One such region is the cytochrome c oxidase subunit I (*COI*) gene, a protein-coding gene in mitochondrial DNA that runs about 648–710 bp in length. The *COI* gene evolves at a moderate pace. This gives it enough variation to tell closely related species apart, yet it stays conserved enough for reliable amplification with universal primers [32]. Sequencing of the COI gene has been widely used to identify and delimit zooplankton species [33,34,35]. Another commonly used marker is the 18S ribosomal RNA (*18S rRNA*) gene. This gene is relatively conserved in its overall structure but has variable regions [36]. Researchers have turned to *18S rRNA* extensively for identifying phytoplankton species [37,38].

During its larval stage, *M. nipponense* feeds mainly on plankton in the water body, including green algae and diatoms. As it grows into the adult stage, it gradually shifts to an omnivorous benthic feeding habit, consuming zooplankton such as aquatic insect larvae [39]. Given the central position of zooplankton in aquatic food webs, this feeding behavior may enhance the prawn’s nutrient profile. Based on these observations, it is hypothesized that certain plankton taxa, particularly those linked to the formation of EAAs and UFAs, may correlate with the nutritional profile of *M. nipponense*. These taxa have not yet been identified, and further work is needed to fill this knowledge gap. In this study, we carried out systematic sampling at ten geographically distinct locations across China: five freshwater sites and five saline–alkaline sites. Water samples, plankton communities, and *M. nipponense* specimens were collected from each site. We then measured the ionic composition and plankton community structure of the water samples, along with the nutritional composition of the prawn specimens. These data allowed us to explore how ionic composition relates to plankton community formation, and how the resulting indicator taxa connect to the nutritional profile of *M. nipponense*. The findings offer scientific evidence for developing artificial techniques to optimize and regulate the nutritional composition of *M. nipponense* in aquaculture settings.

## 2. Materials and Methods

### 2.1. Sample Collection

**Figure 1:**
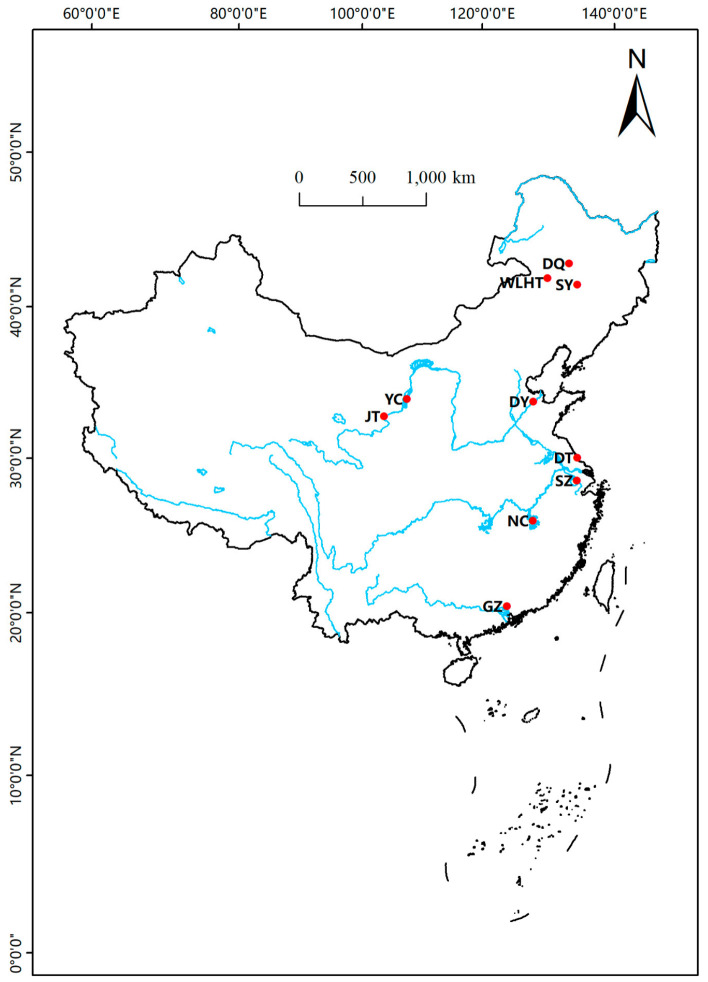
The locations of wild *M. nipponense* populations.

Sampling covered ten geographically distinct locations across China, representing a range of aquatic environments (Figure 1, Table 1). The sampling sites included Daqing (DQ), Songyuan (SY), and Wulanhaote (WLHT) in the northeast; Yinchuan (YC) and Jingtai (JT) in the west; Dongtai (DT), Dongying (DY), and Suzhou (SZ) in the east; and Guangzhou (GZ) and Nanchang (NC) in the south. Table 1 lists the geographic coordinates for each location.

Sampling was conducted in August 2023 under stable environmental conditions, with water temperature ranging from 28.3 to 32.5 °C and dissolved oxygen from 6.8 to 7.3 mg/L. At each location, five sampling points were set up within a 1 km^2^ area: one at the center and one at each of the four cardinal directions. Water, plankton, and *M. nipponense* specimens were collected from these points. Water samples (3 L total) were taken at 9 a.m. from a depth of 0.5 m at each point. These samples were then analyzed to determine salinity, alkalinity, and ionic composition of the water body at each location. Salinity was measured with a calibrated salinity meter. Alkalinity was determined following the SC/T9406-2012 standard [40].

Plankton samples were collected from the water samples using a sterile filtration system (WD-3B, Yinuoji, Nanjing, China) equipped with 3 μm membranes. This process was carried out in triplicate, giving three independent biological replicates. The filters were preserved in DNA later, immediately flash-frozen in liquid nitrogen, and then stored at −80 °C until DNA extraction. *M. nipponense* specimens were collected using five standardized ground cages (80 m in length, 0.4 m in height, 0.5 cm mesh) placed along 1000 m river sections at each location. From each of the five sampling points per location, a total of 300 specimens with both sexes were collected with body weights ranging from 2.5 to 3.5 g. Muscle tissues were dissected from these specimens for subsequent nutrient composition analysis. For each location, an independently pooled sample of 100 g of muscle tissue was used to form one biological replicate, and three such independent biological replicates were prepared. Each biological replicate was then analyzed in triplicate technical replicates.

### 2.2. Ionic Composition Analysis

Water samples were analyzed using an ICS900 ion chromatograph (Thermo Scientific, Waltham, MA, USA). For anion analysis, the following parameters were used: AS23 column, 20 mmol/L KOH as eluent, ASRS 4 mm suppressor set at 50 mA, a flow rate of 1 mL/min, and a column temperature of 20 °C. For cation analysis, the parameters were: CS12 column, 20 mmol/L MSA as eluent, CSRS 4 mm suppressor set at 59 mA, a flow rate of 1 mL/min, and a column temperature of 20 °C. The analytes measured included K^+^, Na^+^, Ca^2+^, Mg^2+^, Cl^−^, SO_4_^2−^, HCO_3_^−^, and CO_3_^2−^.

### 2.3. Nutrient Composition Analysis

Amino acid contents were measured following the national standard GB 5009.124-2016 [41]. Amino acids in muscle tissue were quantified using the acid hydrolysis method. In brief, freeze-dried samples were hydrolyzed at 110 °C for 22 h with 10 mL of 6 mol/L hydrochloric acid. The resulting hydrolysate was then filtered, and amino acid concentrations were determined using an automatic amino acid analyzer (LA8080, Hitachi, Tokyo, Japan). The detection wavelengths were set at 570 nm and 440 nm, and the injection volume was 20 μL. The analysis was carried out with a sulfonic acid cation resin separation column, and the reaction temperature was kept at 135 °C.

Fatty acid contents were determined following the national standard GB 5009.168-2016 [42]. Muscle samples underwent hydrolysis, saponification, and methylation to prepare fatty acid methyl esters (FAMEs). The resulting FAMEs were then analyzed using a gas chromatograph (Agilent 7890A, Agilent Technologies, Santa Clara, CA, USA) equipped with a TG-FAME chromatographic column (50 m × 0.25 mm × 0.20 μm). The inlet temperature was set to 270 °C, and the flame ionization detector was maintained at 280 °C. High-purity nitrogen (N_2_) served as the carrier gas at a flow rate of 0.63 mL/min, with a split ratio of 100:1.

Ash content was determined following the national standard GB 5009.4-2016 [43]. Mixed muscle samples were first placed on an electric heating plate and heated gently until they were fully carbonized and no further smoke was emitted. The carbonized samples were then transferred to a high-temperature furnace and combusted at 550 °C ± 25 °C for 4 h. After combustion, the samples were cooled to room temperature and weighed. This process was repeated until the difference between two consecutive measurements was less than 0.5 mg, indicating that a constant weight had been achieved. Blank measurements were carried out as negative controls to ensure accuracy.

Muscle samples were transferred to centrifuge tubes for astaxanthin extraction. The extraction procedure was as follows: after adding 10 mL of acetone solution and anhydrous sodium sulfate, the mixture was subjected to ultrasonication for 20 min, followed by centrifugation at 8000 rpm for 5 min. The supernatant was collected, and the residue was repeatedly dissolved in acetone and extracted until the solution became colorless. All extraction solutions were pooled and brought to a final volume of 25 mL. A 12.5 mL aliquot of the pooled solution was then transferred to a 15 mL centrifuge tube, dried under nitrogen gas, and redissolved in 2 mL of methanol. The resulting solution was filtered through a 0.45 μm microporous membrane. Astaxanthin quantification was carried out using a liquid chromatograph (HPLC, Agilent 1260, Santa Clara, CA, USA) equipped with a SHISEIDO C30 column. Detection was performed at a wavelength of 450 nm, with an injection volume of 20 μL and a flow rate of 1.0 mL/min.

### 2.4. Plankton Community Analysis

DNA extraction was carried out using the MT062 kit (Yinuoji, Nanjing, China) following the manufacturer’s instructions. For the identification of zooplankton and phytoplankton, two separate PCR amplifications were performed. Each 25 μL reaction contained 0.5 μL of 10 μM forward primer, 0.5 μL of 10 μM reverse primer, 1 μL DNA template, 19.9 μL DEPC water, 2.5 μL 10× PCR supermix, 0.5 μL dNTP mix (10 mM), and 0.1 μL Taq DNA polymerase. The *COI* gene was amplified for zooplankton identification using specific primers (forward: GGWACWGGWTGAACWGTWTAYCCYCC; reverse: TAAACYTCAGGRTGACCRAARAAYCA). The V9 region of the *18S rRNA* gene was amplified for phytoplankton identification using specific primers (forward: TTGTACACACCGCCC; reverse: CCTTCYGCAGGTTCACCTAC). The cycling conditions were as follows: initial denaturation at 95 °C for 3 min; 25 cycles of 95 °C for 20 s, 62 °C for 20 s, and 72 °C for 15 s; followed by a final extension at 72 °C for 5 min. Three negative controls (containing no DNA template) and three positive controls (including an eDNA internal positive standard for phytoplankton and an eDNA internal positive standard for zooplankton from E-genomics, Nanjing, China) were included in all PCR procedures to monitor for contamination. No PCR amplification products were observed in the negative controls, whereas the target products were successfully amplified in the positive controls, confirming the reliability of the reaction system.

PCR products were run on a 2% agarose gel, then purified using VAHTS DNA Clean Beads, and quantified with Qubit™ dsDNA HS Assay Kits (Invitrogen, Waltham, MA, USA). A DNA Library Prep Kit (Vazyme, Nanjing, China) was used to construct the DNA library, which was then sequenced on the Ion Torrent platform with paired-end reads of 2 × 200 bp (ThermoFisher, Waltham, MA, USA). Over 100,000 reads were generated for both the *COI* and *18S rRNA* amplifications. Sequence analysis was carried out using EcoView 3.1 software for filtering, dereplication, merging of paired-end reads, and chimera removal [44]. In sequence quality control, any base whose quality value fell below Q20 was discarded. Sequences were filtered to keep those with lengths between 220 bp and 550 bp. The filtered sequences were clustered into operational taxonomic units (OTUs) using USEARCH (v11.0.667) and aligned against a public database to remove chimeric sequences. Taxonomic identification and annotation of plankton species were performed against the NCBI GenBank database based on the OTUs. The annotation thresholds were set at 97% for species, 95% for genus, 93% for family, 91% for order, and 90% for class. Prior to analysis, the filtered data then went through a low-abundance filtering step. Specifically, OTUs were kept only if they met all three of the following criteria: (1) the sequence count of the OTUs in each sample exceeds 0.0001 times the sequencing depth of that sample; (2) the OTUs are detected in at least two samples; and (3) the total sequence sum of the OTUs is greater than 50. OTU counts were then normalized to relative abundance (relative abundance = OTU reads/total reads) for subsequent analyses, including principal component analysis (PCA), linear discriminant analysis effect size (LEfSe), redundancy analysis (RDA), and alpha and beta diversity analyses.

### 2.5. Statistical Analysis

Multivariate analyses were performed using R software (version 4.0.3) [45]. PCA was conducted to assess community and nutrient composition based on the relative abundance of OTUs. LEfSe was carried out with the R package microeco [46] to identify indicator taxa, using habitat type (freshwater vs. saline–alkaline) as the grouping variable. The following parameters were applied: (1) LDA threshold of 2.0 (default); (2) alpha value of 0.05 for the Kruskal–Wallis test; (3) alpha value of 0.05 for the pairwise Wilcoxon test; (4) scaling factor of 1,000,000 (converting relative abundance to counts per million, CPM). Taxa with an LDA score above 2 and an adjusted *p*-value below 0.05 were considered statistically significant. Raw *p*-values were adjusted for multiple testing using the Benjamini–Hochberg false discovery rate (FDR) method. RDA was performed with the R package vegan [47] to examine correlations between ionic composition and plankton indicator taxa, as well as between plankton indicator taxa and nutritional components, all based on OTU relative abundance. Before analysis, environmental factors and the relative abundance of hypervariable plankton genera were standardized (mean = 0, SD = 1). Multicollinearity was assessed using variance inflation factors (VIFs) calculated with the vegan package (v2.6-4). Variables with a VIF greater than 10 were considered highly collinear and removed one by one. Alpha and beta diversity analyses were also computed with the vegan package [47] from the OTU relative abundance data. NMDS was used to analyze beta diversity based on the Bray–Curtis distance metric. PERMANOVA analysis was performed using the vegan package (v2.6-4). Pearson correlation analysis was used to evaluate associations between ionic composition and plankton indicator taxa, as well as between plankton indicator taxa and nutritional components. All data visualizations were performed with the R package ggplot2 [48]. Statistical significance of differences in nutrient composition across the ten sampling sites was assessed using SPSS 23.0 (IBM, Armonk, NY, USA) with one-way ANOVA, followed by the LSD and Duncan’s multiple range test (*p* < 0.05). Before running the ANOVA, the normality of all variables was checked with the Shapiro–Wilk test, and the homogeneity of variances was verified with Levene’s test. Data are presented as mean ± standard deviation.

## 3. Results

### 3.1. Ionic Composition Analysis in Aquatic Environments

A full analysis of ionic composition was carried out at the ten sampling sites, with major anions (Cl^−^, SO_4_^2−^, HCO_3_^−^, CO_3_^2−^) and cations (K^+^, Na^+^, Ca^2+^, Mg^2+^) measured quantitatively (Table 2). The results showed clear spatial differences in which ions dominated from one location to another. For anions, Cl^−^ was the dominant one at DT, DY, and YC; SO_4_^2−^ represented the first at JT; and HCO_3_^−^ was the main anion at SY, DQ, NC, SZ, GZ, and WLHT. As for cations, Na^+^ was the dominant one at DT, DY, YC, JT, SY, DQ, and GZ, whereas Ca^2+^ was the top cation at WLHT and NC.

### 3.2. Phytoplankton Community Composition Across Sampling Locations

Alkalinity levels at the sampling sites ranged from 0.8 to 6.5 mmol/L, with the highest value found at DY. Salinity, meanwhile, ranged from 0 to 10‰, peaking at JT (Table 1). Based on these measurements, these sampling sites were classified into two types: freshwater (salinity ≤ 2‰ and alkalinity ≤ 3 mmol/L) and saline–alkaline (salinity > 2‰ or alkalinity > 3 mmol/L) [49]. The freshwater group included WLHT, DT, SZ, NC, and GZ. The saline–alkaline group covered DQ, SY, JT, YC, and DY. DT had a salinity of 2‰ and an alkalinity of 2.8 mmol/L, placing it right on the borderline between freshwater and saline–alkaline. However, since its alkalinity fell below 3 mmol/L, it was assigned to the freshwater category.

**Figure 2:**
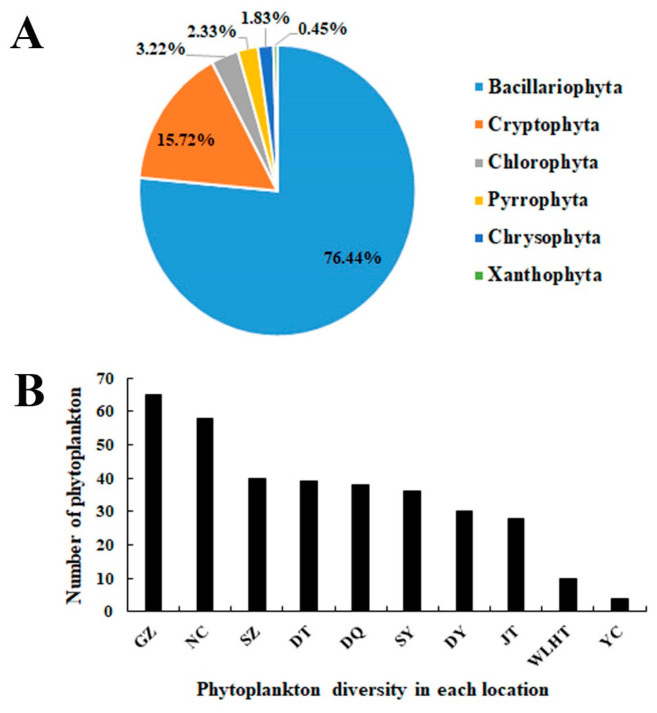
Identification of phytoplankton diversity in the water environment of each location: (**A**) phytoplankton diversity at phylum level; (**B**) phytoplankton number at genus level.

A detailed taxonomic analysis was carried out to characterize the phytoplankton communities at all ten sampling locations. A total of 6 phyla, 44 families, 67 genera, and 97 species were identified. The community showed a clear hierarchical pattern in terms of dominance. Bacillariophyta was the most abundant phylum, representing 76.44% of the sequenced reads, followed by Cryptophyte at 15.72%. The remaining phyla each accounted for relative abundance ranging from 0.45% to 3.22% (Figure 2A).

Genus-level analysis showed notable spatial differences in phytoplankton taxonomic richness across the sampling sites. The highest number of genera (65) was observed in GZ, and the lowest (4) in YC. A comparison between freshwater and saline–alkaline regions further revealed distinct patterns. In freshwater sites, the number of identified genera was as follows: GZ (65 genera), NC (58), SZ (40), DT (39), and WLHT (10). In saline–alkaline sites, the counts were: DQ (38), SY (36), DY (30), JT (28), and YC (4). These results indicate considerable spatial variation in phytoplankton community structure, with freshwater environments generally supporting higher taxonomic richness than saline–alkaline ones.

Genus-level dominance varied with different locations. *Cyclotella* was the dominant genus in DT, DY, YC, SY, and DQ. *Cryptomonas* was predominant in SZ, WLHT, and GZ. In JT, *Chaetoceros* was the most abundant, while in NC, it was *Nitzschia* (Figure 2B).

### 3.3. Zooplankton Community Composition Across Sampling Locations

**Figure 3:**
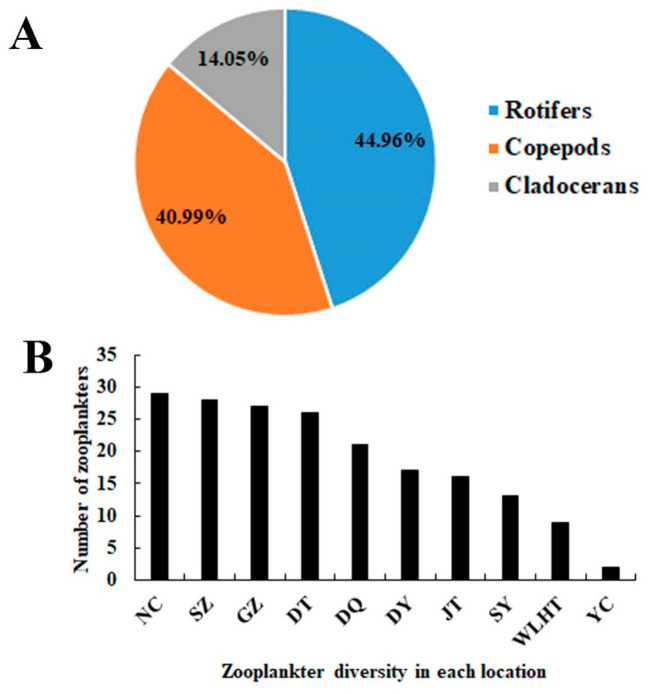
Identification of zooplankter diversity in the water environment of each location: (**A**) zooplankter diversity at phylum level; (**B**) zooplankter number at genus level.

The zooplankton communities at the ten sampling sites were examined systematically. A total of 3 phyla, 20 families, 30 genera, and 34 species were identified. At the phylum level, Rotifera was the largest group (44.96% of the sequenced reads), followed by Copepoda (40.99%) and Cladocera (14.05%) (Figure 3A).

The genus-level results showed clear spatial differences in zooplankton taxonomic richness across sites. The highest number of genera (29) was found in NC, and the lowest (2) in YC. A comparison between freshwater and saline–alkaline regions gave results similar to those seen for phytoplankton: freshwater environments tended to support more genera than saline–alkaline ones. In freshwater sites, the genus counts were 29 (NC), 28 (SZ), 27 (GZ), 26 (DT), and 9 (WLHT). In saline–alkaline sites, the numbers were 21 (DQ), 17 (DY), 16 (JT), 13 (SY), and 2 (YC).

Genus-level dominance also showed clear patterns across locations. *Brachionus* was the predominant genus in YC and JT, while *Moina* dominated in DT and WLHT. For the remaining sites, the most abundant zooplankton genus was as follows: *Limnoithona* in SZ, *Ascomorpha* in GZ, *Cephalodella* in DY, *Arctodiaptomus* in NC, *Keratella* in SY, and *Bosmina* in DQ (Figure 3B).

### 3.4. Nutritional Composition of M. nipponense Across Sampling Locations

The nutritional composition of *M. nipponense* varied greatly across the ten sampling locations (Table 3). Ash content ranged from 1.13 to 1.6 g/100 g, with the highest value found at JT. Astaxanthin content ranged from 0.63 mg/kg (DY) to 2.59 mg/kg (YC). Total amino acids varied between 16.5 g/100 g (SY) and 19.57 g/100 g (JT). Essential amino acids ranged from 4.77 g/100 g to 5.46 g/100 g, with the lowest in YC and the highest in DQ. Total fatty acids ranged from 0.8 g/100 g (DT) to 1.1 g/100 g (DY, DQ, and WLHT). Unsaturated fatty acids reached a maximum of 0.36 g/100 g in SY and a minimum of 0.18 g/100 g in DT.

In saline–alkaline sites, essential amino acid contents were as follows: 5.46 g/100 g (DQ), 5.44 g/100 g (DY), 5.36 g/100 g (SY), 5.07 g/100 g (JT), and 4.77 g/100 g (YC). In freshwater sites, the values were 5.24 g/100 g (GZ), 5.17 g/100 g (WLHT), 5.16 g/100 g (DT), 5.07 g/100 g (NC), and 4.92 g/100 g (SZ). For unsaturated fatty acids, saline–alkaline sites were 0.36 g/100 g (SY), 0.35 g/100 g (DY), 0.33 g/100 g (DQ), 0.29 g/100 g (JT), and 0.19 g/100 g (YC). In freshwater sites, the values were 0.32 g/100 g (WLHT), 0.25 g/100 g (NC and GZ), 0.23 g/100 g (SZ), and 0.18 g/100 g (DT). These results indicated that *M. nipponense* from the saline–alkaline environments of DQ, DY, and SY had better nutritional quality, especially in terms of essential amino acids and unsaturated fatty acids, than those from the other locations.

### 3.5. Biodiversity of Plankton Across Sampling Locations

**Figure 4:**
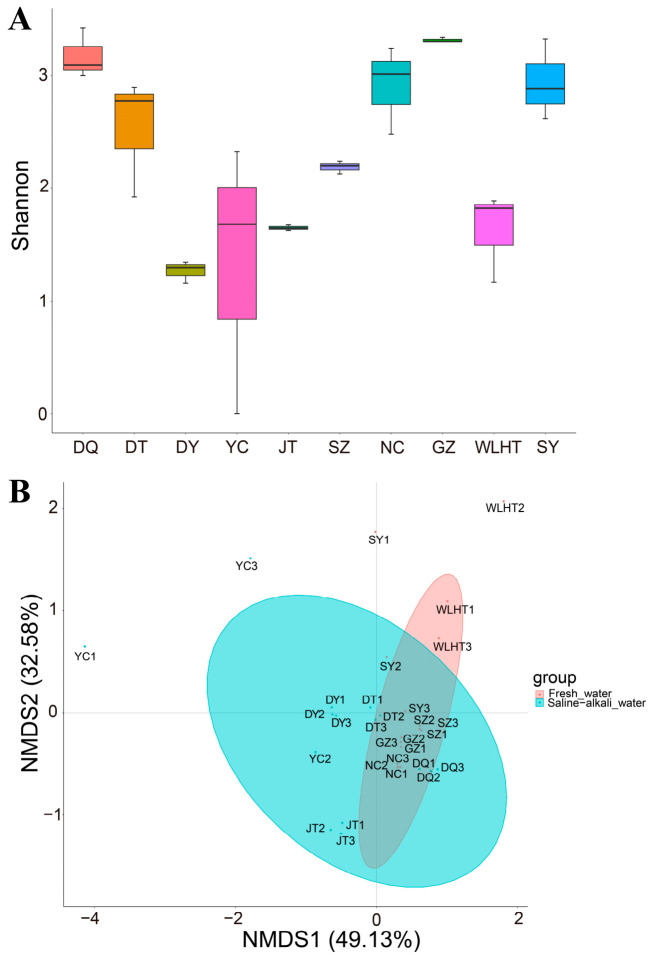
Alpha and beta diversity analysis of plankton: (**A**) alpha diversity analysis of plankton by Shannon analysis; (**B**) beta diversity analysis of plankton by NMDS analysis.

Plankton biodiversity at the ten sampling sites was assessed using alpha and beta diversity metrics. Alpha diversity, measured by the Shannon index, varied considerably across locations, with values ranging from 1.26 to 3.31. The index values were 2.53 (DT), 2.18 (SZ), 1.62 (WLHT), 1.33 (YC), 1.64 (JT), 3.31 (GZ, the highest), 1.26 (DY, the lowest), 3.17 (DQ), 2.94 (SY), and 2.91 (NC) (Figure 4A).

Beta diversity analysis showed that plankton species composition did not fully diverge between saline–alkaline and freshwater ecosystems. However, the PERMANOVA analysis gave a *p*-value of 1 × 10^−4^ between the two habitats, suggesting that the two habitats did differ in terms of plankton community. The species distribution patterns seen in DY (*p* < 0.001) and JT (*p* < 0.001) differed clearly from those at all other sampling sites, pointing to unique ecological conditions in these two habitats (Figure 4B).

### 3.6. Identification of Plankton Indicator Taxa Distinguishing Freshwater and Saline–Alkaline Water Regions

**Figure 5:**
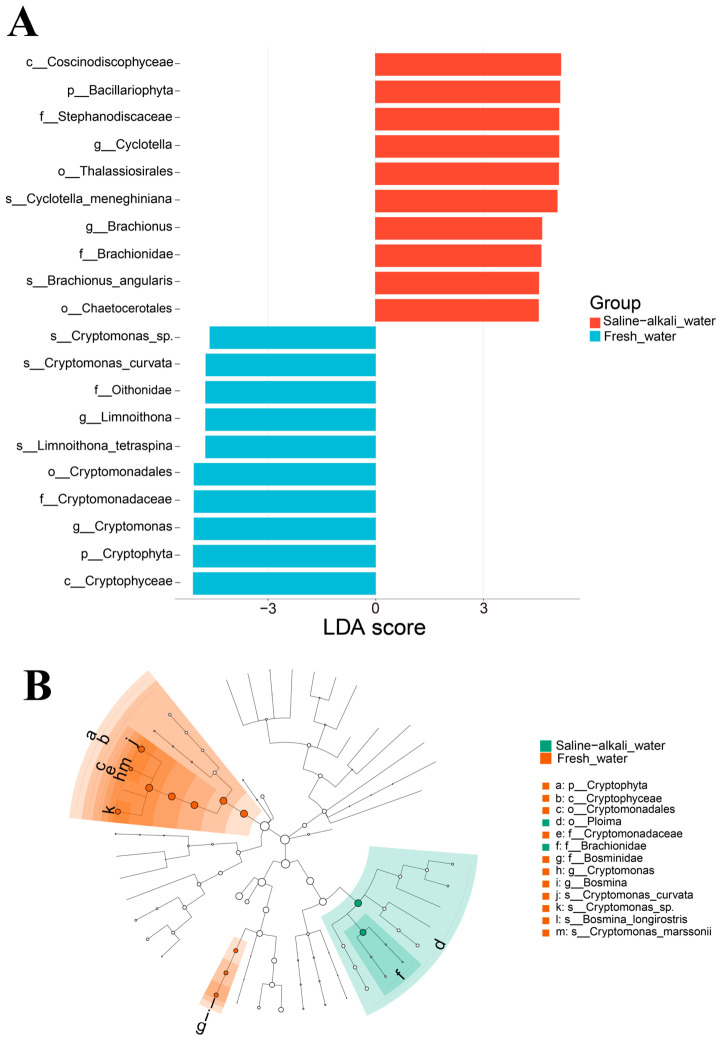
Identification of the plankton indicator taxa between the freshwater resources and saline–alkaline water resources: (**A**) the 10 plankton indicator taxa with the greatest difference in the freshwater resources (blue color) and saline–alkaline water resources (red color), analyzed by the LEfSe; (**B**) cladogram of LEfSe.

Plankton indicator taxa and representative microorganisms were identified using LEfSe, based on relative read abundance, to distinguish the taxa between freshwater and saline–alkaline water ecosystems. The analysis picked out 20 indicator taxa that showed the largest differences in relative abundance between the two habitat types. At higher taxonomic levels, the most distinctive indicator taxa linked to saline–alkaline waters were Coscinodiscophyceae (class level), Bacillariophyta (phylum level), and Stephanodiscaceae (family level). On the other hand, Cryptophyceae (class level), Cryptophyta (phylum level), and Cryptomonas (genus level) were indicator taxa associated with freshwater environments (Figure 5A).

At the genus level, six indicator taxa showed clear differences in relative abundance between the two habitat types. *Cyclotella*, *Brachionus*, and *Chaetoceros* were associated with saline–alkaline waters, while *Arctodiaptomus*, *Cryptomonas*, and *Limnoithona* were linked to freshwater ecosystems (Figure 5B).

### 3.7. Correlation Analysis Between Ionic Composition and Plankton Indicator Taxa

**Figure 6:**
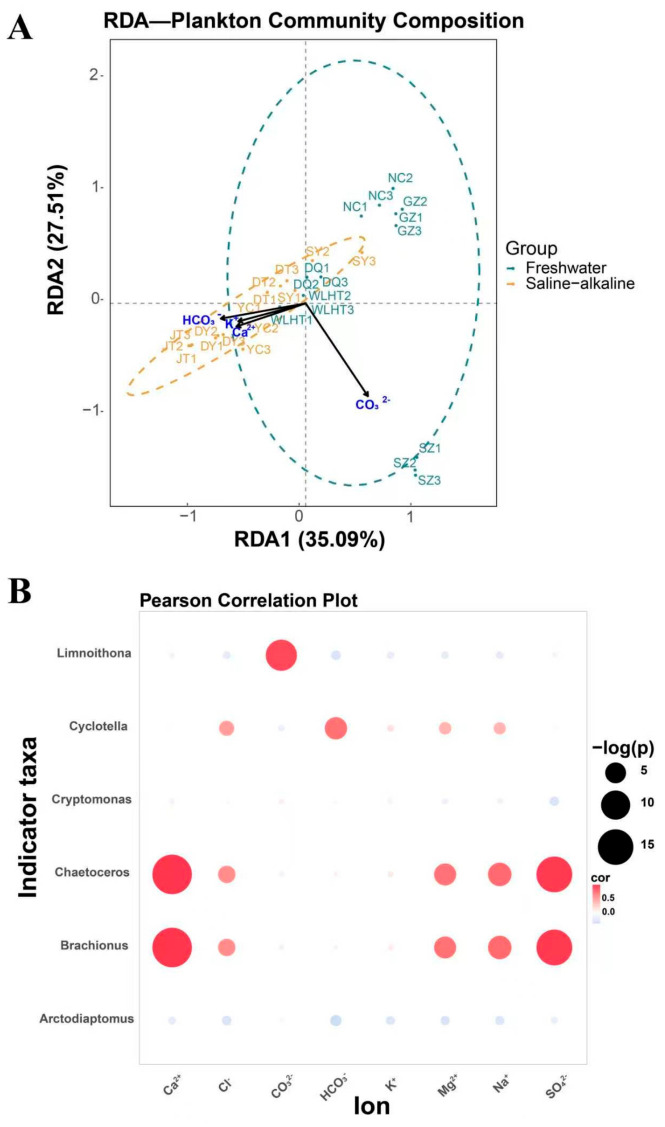
Identification of the correlations between ionic compositions and plankton indicator taxa: (**A**) RDA for the relationships between ionic compositions and plankton indicator taxa; (**B**) correlation between ionic compositions and the richness of indicator taxa. Colors represent the coefficient of determination (R^2^), and point sizes indicate the significance level. Red indicates a positive correlation, and blue indicates a negative correlation.

RDA was carried out to examine the relationships between ionic composition and plankton community richness in water samples from the ten sites. After removing highly collinear variables (Cl^−^, Na^+^, Mg^2+^, SO_4_^2−^) step by step, the final model included four variables: HCO_3_^−^, CO_3_^2−^, K^+^, and Ca^2+^. All had VIF values below 2 (HCO_3_^−^: 1.26, CO_3_^2−^: 1.15, K^+^: 1.18, Ca^2+^: 1.04), indicating no major multicollinearity. The permutation test for the RDA model gave a value of 0.001, and the ratio of constrained inertia to total inertia was 33.64%. The results showed that, except for SY, K^+^, Ca^2+^, and HCO_3_^−^ were positively correlated with plankton community richness in saline–alkaline water regions. In contrast, CO_3_^2−^ was positively correlated with plankton community richness at the SZ site (Figure 6A).

Pearson correlation analysis showed that Na^+^, Mg^2+^, Ca^2+^, and SO_4_^2−^ were positively correlated with the presence of *Chaetoceros* and *Brachionus* (*p* < 0.05). HCO_3_^−^ was positively correlated with the presence of *Cyclotella* (*p* < 0.05), and CO_3_^2−^ was positively correlated with the presence of *Limnoithona* (*p* < 0.05) (Figure 6B).

### 3.8. Correlation Analysis Between Plankton Indicator Taxa and Nutritional Components of M. nipponense

**Figure 7:**
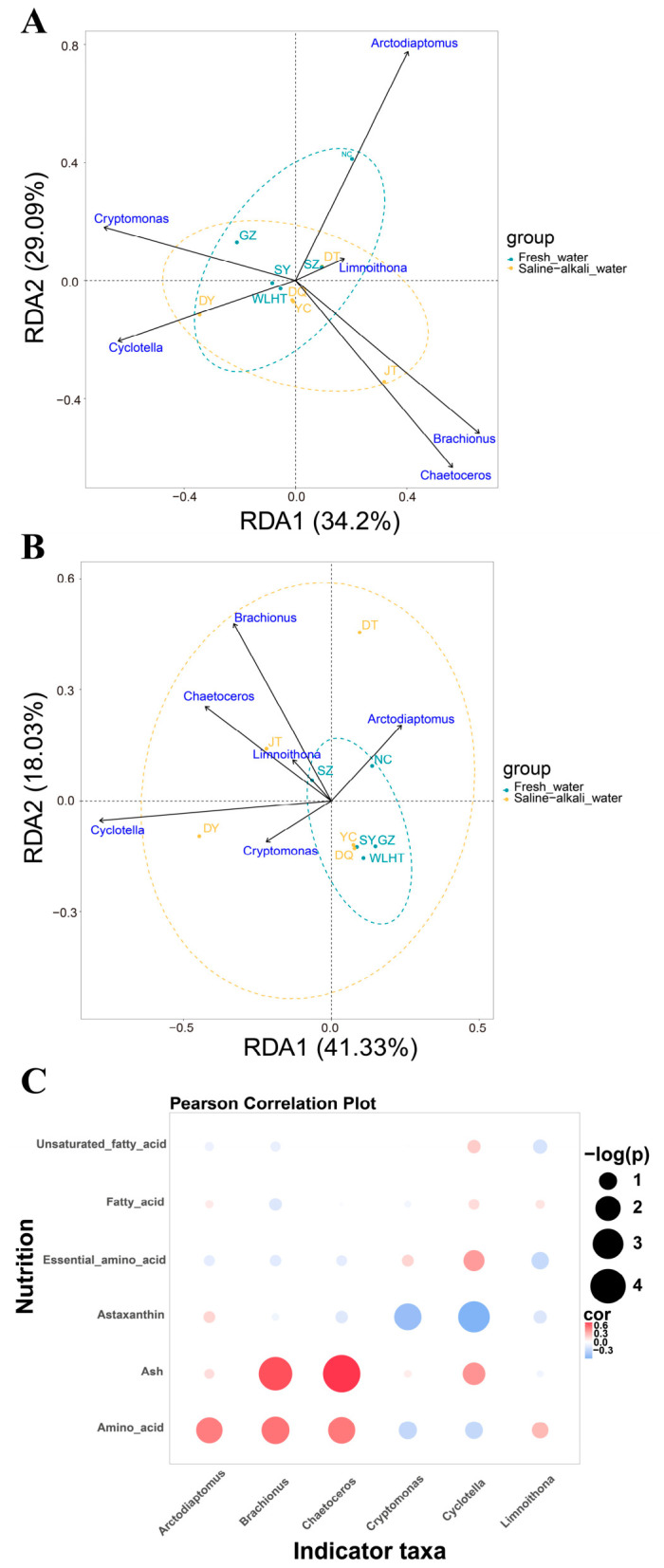
Correlations between plankton indicator taxa and nutrient values: (**A**) RDA for the associations between plankton indicator taxa and amino acid nutrient values; (**B**) RDA for the associations between plankton indicator taxa and fatty acid nutrient values; (**C**) correlation between plankton indicator taxa and nutrient compositions. Colors represent the coefficient of determination (R^2^), and point sizes indicate the significance level. Red indicates a positive correlation, and blue indicates a negative correlation.

RDA was used to explore the relationships between plankton indicator taxa and the nutritional composition of *M. nipponense* collected from the ten sites. All VIF values were below 5 (*Cryptomonas*: 4.50, *Cyclotella*: 4.06, *Acanthocyclops*: 2.34, *Limnoithona*: 2.31, *Chaetoceros*: 1.49, *Brachionus*: 1.16), indicating no significant multicollinearity among the selected genera. For amino acids, the permutation test for the RDA model gave a value of 0.001, and the ratio of constrained inertia to total inertia was 45.91%. For fatty acids, the permutation value was 0.002, and the constrained-to-total inertia ratio was 40.03%. The RDA results showed that the amino acid and fatty acid profiles of *M. nipponense* did not clearly differ between saline–alkaline and freshwater sites. However, certain plankton indicator taxa were significantly correlated with the nutritional composition of *M. nipponense* in specific locations. For example, *Arctodiaptomus* showed a strong positive correlation with the amino acid composition of *M. nipponense* from NC, while *Brachionus* and *Chaetoceros* were positively correlated with that from JT (Figure 7A). Additionally, *Limnoithona*, *Chaetoceros*, and *Brachionus* were positively correlated with the fatty acid composition of *M. nipponense* from SZ and JT, but negatively correlated with that from SY, WLHT, GZ, YC, and DQ (Figure 7B).

Pearson correlation analysis showed that *Arctodiaptomus*, *Brachionus*, and *Chaetoceros* were positively correlated with total amino acid content in *M. nipponense* (*p* < 0.05). In contrast, the presence of *Cryptomonas* and *Cyclotella* in the water column was linked to reduced astaxanthin synthesis (*p* < 0.05). Additionally, ash content in *M. nipponense* was significantly higher in waters where *Brachionus* and *Chaetoceros* were the dominant plankton genera (*p* < 0.05) (Figure 7C).

## 4. Discussion

Water quality in aquatic environments can be grouped into several hydrochemical types, such as carbonate, sulfate, chloride, calcium, magnesium, and sodium. In waters with low mineralization, the carbonate type tends to dominate [50]. As mineralization rises, sulfate and chloride types become more common. In coastal mudflat settings, the chloride type is usually the main one [51]. A detailed measurement of ionic composition showed clear spatial differences across the sampled sites, pointing to regional variations in geology and human activities. In this study, *M. nipponense* was mainly collected from waters where Cl^−^ or HCO_3_^−^ were the dominant anions and Na^+^ was the dominant cation.

The water environment and food sources play a role in shaping the nutritional composition of *Macrobrachium* species [52]. Earlier studies have reported large differences in the nutritional content of *M. nipponense* from different geographic areas [53,54,55,56]. In addition, wild populations tend to have better nutritional quality than farmed ones [54,57]. In the present study, the nutritional components of *M. nipponense* also varied greatly among the ten sampling sites, which matches what has been seen before. This suggests that food sources in the water environment are associated with the nutritional formation of *M. nipponense*. Notably, prawns from DQ, DY, and SY (areas with saline–alkaline water) showed higher nutritional value, especially in terms of essential amino acids and unsaturated fatty acids, both key indicators of aquatic product quality. Therefore, it is worth investigating the plankton in these water environments to develop artificial methods for improving the nutritional value of *M. nipponense*.

Plankton has many functions in aquaculture and paleolimnology. It serves as a main food source for fish in natural waters and has a strong influence on fish production and nutrient composition [12]. In this study, plankton richness was measured across ten water environments in China. At the phylum level, rotifers were the most abundant zooplankton, and Bacillariophyta were the most abundant phytoplankton. Rotifers are sensitive to water quality changes, which makes them useful bioindicators for assessing water conditions [58]. High numbers of *Brachionus* often point to organic pollution in a water body [59,60]. In this study, both phytoplankton and zooplankton generally had higher species richness in freshwater areas than in saline–alkaline ones, suggesting that saline–alkaline conditions have negative effects on plankton community richness. Notably, 65 phytoplankton genera were found at GZ, but only 4 at YC. This large difference points to major ecological differences between the two sites, and further work is needed to investigate the possible reasons. Ionic composition in water is known to shape plankton abundance. Changes in which ions are present can keep some species out while helping others grow [9]. Earlier studies have offered plausible reasons for this, such as high levels of dissolved inorganic carbon in carbonate-rich waters, high temperatures, and cyanobacteria blooms [61,62,63]. These findings fit with the observation that high salinity and alkalinity have stress on zooplankton and phytoplankton communities. For example, chloride, a key ion that drives salinity, has been shown to have a negative link with species occurrence. As salinity increases, the number of species and overall biodiversity decrease, with less tolerant species being excluded and specific life stages being acutely affected [9,10]. Previous studies have also shown that ionic composition regulates plankton communities in different water bodies. In Tiga Lake, Nigeria, Ca^2+^, Na^+^, and K^+^ were reported to positively regulate zooplankton abundance, while NO_3_^−^ and PO_4_^3−^ had negative effects [64]. In carbonate-rich waters, higher levels of dissolved inorganic carbon and carbonate ions (CO_3_^2−^) have been shown to boost certain phytoplankton (especially cyanobacteria), while at the same time holding back other groups [61]. Similarly, sulfate ions (SO_4_^2−^) have been found to strongly shape plankton community structure in saline–alkaline lakes, favoring cyanobacteria but reducing diatoms and green algae [63]. In the present study, the abundance of *Chaetoceros* and *Brachionus* in saline–alkaline waters is associated with levels of K^+^, Ca^2+^, and HCO_3_^−^ (except at SY). At the SZ site, plankton community richness increased with the level of CO_3_^2−^. These findings suggested that ionic composition was linked to plankton community structure, though this relationship may be partly confounded by how the sampling sites were grouped based on their water chemistry.

In this study, based on relative sequence abundance from metabarcoding data, six indicator taxa at the genus level showed clear differences in diversity between water samples from saline–alkaline and freshwater regions. Specifically, *Cyclotella*, *Brachionus*, and *Chaetoceros* were associated taxa in saline–alkaline regions, while *Arctodiaptomus*, *Cryptomonas*, and *Limnoithona* were associated taxa in freshwater regions. These six were identified as key indicators of environmental differences between the two types of sampling regions. Pearson correlation analysis showed that Na^+^, Mg^2+^, Ca^2+^, and SO_4_^2−^ were positively linked to the presence of *Chaetoceros* and *Brachionus* (*p* < 0.05). Meanwhile, HCO_3_^−^ was positively associated with *Cyclotella*, and CO_3_^2−^ with *Limnoithona* (*p* < 0.05).

Small planktonic species are a major food source in aquaculture, especially for fish larvae and shellfish [65]. These organisms naturally carry key nutrients like proteins, lipids, carbohydrates, vitamins, minerals, fatty acids, and amino acids. These nutrients are essential for the growth and health of farmed species [66]. Adding certain plankton to diets has been shown to improve nutritional value compared to using formulated feeds alone. Zooplankton such as rotifers (*Brachionus plicatilis*) and brine shrimp (*Artemia salina*) supply digestive enzymes like trypsin and pepsin, which are crucial for the development of young aquatic animals [67]. Zooplankton are especially important in early life stages, when artificial diets cannot fully meet the nutritional needs of larvae. In addition, cladocerans like *Moina* sp. and *Daphnia* sp. are good protein sources that are easy to culture and harvest [68]. Fish larvae and shellfish also need enough carbohydrates in their diets [69,70,71]. The present study showed that the identified plankton indicator taxa had possible links to the nutrient composition of *M. nipponense*. Specifically, *Arctodiaptomus*, *Brachionus*, and *Chaetoceros* may be positively related to amino acid formation, while *Limnoithona*, *Chaetoceros*, and *Brachionus* were positively correlated with fatty acid formation. Two main mechanisms could explain these effects. First, the metabolic activities of these plankton (such as photosynthesis and respiration) can noticeably change the pH and chemical makeup of the surrounding water, which in turn affects how much essential iron and trace metals are available to aquatic organisms [72]. Second, these plankton serve as a direct food source, supplying energy and nutrients that shape the nutrient profile of *M. nipponense* [67].

In this study, certain plankton taxa were found to be linked to better nutrient composition in *M. nipponense*. However, water, plankton, and prawn samples were all collected in a single season (August), which may limit how well the findings on plankton communities can be generalized. Given the small number of sampling sites, the correlational nature of the study design, and the presence of several confounding geographic variables, the role of these plankton in improving the muscle quality of *M. nipponense* needs further investigation and confirmation.

In summary, this study found that *M. nipponense* lives mostly in waters where Cl^−^ or HCO_3_^−^ are the main anions and Na^+^ is the main cation. Nutrient composition varied clearly among populations from different locations, suggesting a link between food sources in the water and the nutritional compositions of this species. Notably, prawns from DQ, DY, and SY, which are areas with saline–alkaline water, had better nutrient profiles than those from other sites, with higher levels of essential amino acids and unsaturated fatty acids. This points to a possible connection between certain plankton communities and the nutritional quality of *M. nipponense*. Six indicator taxa were identified that differed clearly in relative abundance between saline–alkaline and freshwater areas. *Cyclotella*, *Brachionus*, and *Chaetoceros* were linked to saline–alkaline regions, while *Arctodiaptomus*, *Cryptomonas*, and *Limnoithona* were linked to freshwater regions. These six genera are predicted to relate to the nutrient profiles of *M. nipponense*. RDA and Pearson correlation showed that, except at the SY site, the abundance of *Chaetoceros* and *Brachionus* in saline–alkaline waters was associated with the levels of K^+^, Ca^2+^, and HCO_3_^−^. At the SZ site, plankton community richness was linked to CO_3_^2−^. Also, the identified plankton indicator taxa were correlated with the nutritional composition of specific *M. nipponense* populations. This study highlights how ionic composition relates to the formation of indicator taxa in both saline–alkaline and freshwater environments, and how these taxa may shape the nutrient profiles of *M. nipponense*. These findings lay the groundwork for developing artificial methods to improve the nutritional quality of *M. nipponense*, especially by boosting essential amino acids and unsaturated fatty acids, thus raising its value as a food source.

## Figures and Tables

**Table 1:** The coordinates, saline and alkaline concentration of ten locations.

Location	Coordinate	River	Salinity (‰)	Alkalinity (mmol)
DQ	124°11′35″; 46°47′37″	Lianhuanhu	2	5.1
SY	124°29′35″; 45°8′45″	Chaganhu	1	3.3
WLHT	121°38′6″; 46°6′3″	Chaersen Reservoir	0	1.1
YC	106°24′23″; 38°25′46″	Yellow River	1	3.7
JT	104°18′8″; 37°11′12″	Yellow River	10	2.4
DY	117°58′38″; 37°23′17″	Coastal mudflat	3	6.5
DT	120°51′58″; 32°49′36″	Coastal mudflat	2	2.8
SZ	120°27′42″; 31°13′15″	Taihu Lake	0	0.8
NC	116°21′13″; 28°56′52″	Poyang Lake	0	0.8
GZ	113°33′11″; 23°17′12″	Pearl River	0	0.8

**Table 2:** Identification of the ionic components in the water environments from different locations.

Location	Cl^−^ (mg/L)	SO_4_^2−^ (mg/L)	HCO_3_^−^ (mg/L)	CO_3_^2−^ (mg/L)	K^+^ (mg/L)	Na^+^ (mg/L)	Ca^2+^ (mg/L)	Mg^2+^ (mg/L)
DT	388	50.8	247	0	19.6	288	35.1	35.4
DY	735	159	660	0	10.2	710	36.2	104
SY	26.6	27.8	256	0	2.47	58.4	29.0	16.4
DQ	92.2	24.2	392	0	3.67	187	18.2	20.2
JT	814	1.34 × 10^3^	268	0	10.7	1.09 × 10^3^	148	149
YC	540	390	303	0	22.2	463	14.9	79.7
NC	4.73	9.74	39.8	0	2.41	4.40	13.5	2.13
SZ	44.0	49.7	52.1	9.77	3.55	37.4	21.6	8.78
GZ	48.8	31.2	67.9	0	5.62	31.5	31.2	3.95
WLHT	6.45	11.0	75.5	0	0.943	7.75	24.7	5.01

**Table 3:** Identification of nutritional components in *M. nipponense* from different locations.

	Ash (g/100 g)	Astaxanthin (mg/kg)	Total AA (g/100 g)	Essential AA (g/100 g)	Total FA (g/100 g)	Unsaturated FA (g/100 g)
DY	1.5 ± 0 ^e^	0.63 ± 0.02 ^a^	16.77 ± 0.15 ^a^	5.44 ± 0.07 ^de^	1.1 ± 0.1 ^c^	0.35 ± 0.014 ^fg^
SY	1.2 ± 0 ^b^	2.17 ± 0.01 ^g^	16.5 ± 0.36 ^a^	5.39 ± 0.21 ^de^	1.07 ± 0.12 ^c^	0.36 ± 0.008 ^g^
DQ	1.3 ± 0 ^c^	1.66 ± 0.03 ^e^	18.43 ± 0.4 ^c^	5.46 ± 0.09 ^e^	1.1 ± 0.1 ^c^	0.33 ± 0.003 ^ef^
JT	1.6 ± 0 ^f^	1.38 ± 0.05 ^d^	19.57 ± 0.35 ^d^	5.07 ± 0.16 ^bc^	1.0 ± 0.1 ^c^	0.29 ± 0.007 ^d^
YC	1.13 ± 0.06 ^a^	2.59 ± 0.06 ^h^	16.53 ± 0.31 ^a^	4.77 ± 0.19 ^a^	0.83 ± 0.06 ^ab^	0.19 ± 0.007 ^a^
DT	1.3 ± 0 ^c^	2.09 ± 0.09 ^g^	17.37 ± 0.35 ^b^	5.16 ± 0.18 ^bcd^	0.8 ± 0.1 ^a^	0.18 ± 0.004 ^a^
NC	1.4 ± 0 ^d^	2.01 ± 0.04 ^f^	19.5 ± 0.1 ^d^	5.07 ± 0.07 ^bc^	1.03 ± 0.06 ^c^	0.25 ± 0.017 ^c^
SZ	1.3 ± 0 ^c^	1.29 ± 0.04 ^bc^	18.83 ± 0.21 ^c^	4.92 ± 0.17 ^ab^	1.03 ± 0.06 ^c^	0.23 ± 0.012 ^b^
GZ	1.3 ± 0 ^c^	1.22 ± 0.03 ^b^	17.27 ± 0.15 ^b^	5.24 ± 0.19 ^cde^	0.97 ± 0.06 ^bc^	0.25 ± 0.005 ^c^
WLHT	1.33 ± 0.06 ^c^	1.33 ± 0.05 ^cd^	16.37 ± 0.21 ^a^	5.17 ± 0.12 ^bcd^	1.1 ± 0.1 ^c^	0.32 ± 0.011 ^e^

The letters indicated significant differences in nutrient components among the different locations (*p* < 0.05).

## Data Availability

All data have been described in the manuscript.
